# Robinson-Foulds Supertrees

**DOI:** 10.1186/1748-7188-5-18

**Published:** 2010-02-24

**Authors:** Mukul S Bansal, J Gordon Burleigh, Oliver Eulenstein, David Fernández-Baca

**Affiliations:** 1 The Blavatnik School of Computer Science, Tel Aviv University, Tel Aviv 69978, Israel; 2 Department of Computer Science, Iowa State University, Ames, IA 50011, USA; 3 Department of Biology, University of Florida, Gainesville, FL 32611, USA

## Abstract

**Background:**

Supertree methods synthesize collections of small phylogenetic trees with incomplete taxon overlap into comprehensive trees, or supertrees, that include all taxa found in the input trees. Supertree methods based on the well established Robinson-Foulds (RF) distance have the potential to build supertrees that retain much information from the input trees. Specifically, the RF supertree problem seeks a binary supertree that minimizes the sum of the RF distances from the supertree to the input trees. Thus, an RF supertree is a supertree that is consistent with the largest number of clusters (or clades) from the input trees.

**Results:**

We introduce efficient, local search based, hill-climbing heuristics for the intrinsically hard RF supertree problem on rooted trees. These heuristics use novel non-trivial algorithms for the SPR and TBR local search problems which improve on the time complexity of the best known (naïve) solutions by a factor of Θ(*n*) and Θ(*n*^2^) respectively (where *n *is the number of taxa, or leaves, in the supertree). We use an implementation of our new algorithms to examine the performance of the RF supertree method and compare it to matrix representation with parsimony (MRP) and the triplet supertree method using four supertree data sets. Not only did our RF heuristic provide fast estimates of RF supertrees in all data sets, but the RF supertrees also retained more of the information from the input trees (based on the RF distance) than the other supertree methods.

**Conclusions:**

Our heuristics for the RF supertree problem, based on our new local search algorithms, make it possible for the first time to estimate large supertrees by directly optimizing the RF distance from rooted input trees to the supertrees. This provides a new and fast method to build accurate supertrees. RF supertrees may also be useful for estimating majority-rule(-) supertrees, which are a generalization of majority-rule consensus trees.

## Introduction

Supertree methods provide a formal approach for combining small phylogenetic trees with incomplete species overlap in order to build comprehensive species phylogenies, or supertrees, that contain all species found in the input trees. Supertree analyses have produced the first family-level phylogeny of flowering plants [1] and the first phylogeny of nearly all extant mammal species [2]. They have also enabled phylogenetic analyses using large-scale genomic data sets in bacteria, across eukaryotes, and within plants [3,4] and have helped elucidate the origin of eukaryotic genomes [5]. Furthermore, supertrees have been used to examine rates and patterns of species diversification [1,2], to test hypotheses regarding the structure of ecological communities [6], and to examine extinction risk in current species [7].

Although supertrees can support large-scale evolutionary and ecological analyses, there are still numerous concerns about the performance of existing supertree methods (e.g., [8-14]). In general, an effective supertree method must accurately estimate phylogenies from large data sets in a reasonable amount of time while retaining much of the phylogenetic information from the input trees.

By far the most commonly used supertree method is matrix representation with parsimony (MRP), which works by solving the parsimony problem on a binary matrix representation of the input trees [15,16]. While the parsimony problem is NP-hard, MRP can take advantage of fast and effective hill-climbing heuristics implemented in PAUP* or TNT (e.g., [17-19]). MRP heuristics often perform well in analyses of both simulated and empirical data sets (e.g., [20-22]); however, there are numerous criticisms of MRP. For example, MRP shows evidence of biases based on the shape and size of input trees [8,11], and MRP supertrees may contain relationships that are not supported by any of the input trees [9,12]. Furthermore, it is unclear if or why minimizing the parsimony score of a matrix representation of input trees is a good optimality criterion or should produce accurate supertrees.

Since evolutionary biologists rarely, if ever, know the true relationships for a group of species, it is difficult to assess the accuracy of supertree, or any phylogenetic, methods. One approach to evaluate the accuracy of supertrees is with simulations (e.g., [20,21]). However, simulations inherently simplify the true processes of evolution, and it is unclear how well the performance of a phylogenetic method in simulations corresponds to its performance with empirical data. Perhaps a more useful way to define the accuracy of a supertree method is to quantify the amount of phylogenetic information from the input trees that is retained in the supertree. Ideally, we want the supertree to reflect the input tree topologies as much as possible. This suggests that the supertree objective should directly evaluate the similarity of the supertree to the input trees (e.g., [11,23,24]).

Numerous metrics exist to measure the similarity of input trees to a supertree, and the Robinson-Foulds (RF) distance metric [25] is among the most widely used. In fact, numerous studies have evaluated the performance of supertree methods, including MRP, by measuring the RF distance between collections of input trees and the resulting supertrees (e.g., [11,20,21]). The RF supertree problem seeks a binary supertree that minimizes the sum of the RF distances between every rooted input tree and the supertree. The intuition behind seeking a *binary *supertree is that, in this setting, minimizing the RF distance is equivalent to maximizing the number of clusters (or clades) that are shared by the supertree and the input trees. Thus, an RF supertree is a supertree that is consistent with the largest number of clusters from the input trees. Unfortunately, as with MRP, computing RF supertrees is NP-hard [26]. In this work, we describe efficient hill-climbing heuristics to estimate RF supertrees. These heuristics allow the first large-scale estimates of RF supertrees and comparisons of the accuracy of RF supertrees to other commonly used supertree methods.

The RF distance metric between two rooted trees is defined to be a normalized count of the symmetric difference between the sets of clusters of the two trees. In the supertree setting, the input trees will often have only a strict subset of the taxa present in the supertree. Thus, a high RF distance between an input tree and a supertree does not necessarily correspond to conflicting evolutionary histories; it can also indicate incomplete phylogenetic information. Consequently, in order to compute the RF distance between an input tree which has only a strict subset of the taxa in the supertree, we first restrict the supertree to only the leaf set of the input tree. This adapted version of the RF distance is not a metric, or even a distance measure (mathematically speaking). However, for convenience, we will refer to this adapted version of the RF distance metric using the same name.

### Previous work

Supertree methods are a generalization of consensus methods, in which all the input trees have the same leaf set. The problem of finding an optimal median tree under the RF distance in such a consensus setting is well-studied. In particular, it is known that the majority-rule consensus of the input trees must be a median tree [27], and it can be found in polynomial time. On the other hand, finding the optimum *binary *median tree, i.e. an RF supertree, in the consensus setting is NP-hard [26]. This implies that computing an RF supertree in general is NP-hard as well.

Our definition of RF distance between two trees where one has only a strict subset of the taxa in the other, corresponds to the distance measure used to define "majority-rule(-) supertrees" by Cotton and Wilkinson [28]. This definition restricts the larger tree to only the leaf set of the smaller tree before evaluating the RF distance. Majority-rule(-) supertrees are defined to be the strict consensus of all the optimal median trees under the RF distance. These median trees are defined similarly to RF supertrees, except that RF supertrees must be binary while the median trees can be non-binary. In general, majority-rule supertrees [28], in both their (-) and (+) variants, seek to generalize the majority-rule consensus. Indeed, majority-rule supertrees have been shown to have several desirable properties reminiscent of majority-rule consensus trees [29]. Although majority-rule supertrees and RF supertrees are both based on minimizing RF distance, they represent two different approaches to supertree construction. In particular, the RF supertree method seeks a supertree that is consistent with the largest number of clusters (clades) from the input trees, while majority-rule supertrees do not. Nevertheless, as we discuss later, RF supertrees could be used as a starting point to estimate majority-rule(-) supertrees.

The RF distance between two trees on the same size *n *leaf set, with leaves labeled by integers {1, ..., *n*}, can be computed in *O*(*n*) time [30]. In fact, an (1 + ϵ)-approximate value of the RF distance can be computed in sub-linear time, with high probability [31].

In the case of unrooted trees, the RF distance metric is sometimes also known as the splits metric (e.g., [32]). The supertree analysis package Clann [23] provides heuristics that operate on unrooted trees and attempt to maximize the number of splits shared between the input trees and the inferred supertree. This method is called the "maximum splits-fit" method.

### Local Search

We use a heuristic approach for the RF supertree problem. Local search is the basis of effective heuristics for many phylogenetic problems. These heuristics iteratively search through the space of possible supertrees guided, at each step, by solutions to some local search problem. More formally, in these heuristics, a *tree graph *(see [32,33]) is defined for the given set of input trees and some fixed tree edit operation. The node set of this tree graph represents the set of all supertrees on the given input trees. An edge is drawn between two nodes exactly if the corresponding trees can be transformed into each other by one tree edit operation. In our setting, the *cost *of a node in the graph is the RF distance between the supertree represented by that node and the given input trees. Given an initial node in the tree graph, the heuristic's task is to find a maximal-length path of steepest descent in the cost of its nodes and to return the last node on such a path. This path is found by solving the *local search problem *at every node along the path. The local search problem is to find a node with the minimum cost in the neighborhood of a given node. The neighborhood is defined by some tree edit operation, and hence, the time complexity of the local search problem depends on the tree edit operation used.

Two of the most extensively used tree edit operations for supertrees are rooted Subtree Prune and Regraft (SPR) [33-35] and rooted Tree Bisection and Reconnection (TBR) [22,33,34]. The best known (naïve) algorithms for the SPR and TBR local search problems for the RF supertree problem require *O*(*kn*^3^) and *O*(*kn*^4^) time respectively, where *k *is the number of input trees, and *n *is the number of leaves in the supertree solution.

### Our Contribution

We describe efficient hill-climbing heuristics for the RF supertree problem. These heuristics are based on novel non-trivial algorithms that can solve the corresponding local search problems for both SPR and TBR in *O*(*kn*^2^) time, yielding speed-ups of Θ(*n*) and Θ(*n*^2^) over the best known solutions respectively. These new algorithms are inspired by fast local search algorithms for the gene duplication problem [36,37]. Note that while the supertree itself must be binary, our algorithms work even if the input trees are not. We also examine the performance of the RF supertree method using four published supertree data sets, and compare its performance with MRP and the triplet supertree method [38]. We demonstrate that the new algorithms enable RF supertree analyses on large data sets and that the RF supertree method outperforms other supertree methods in finding supertrees that are most similar to the input trees based on the RF distance metric.

## Basic Notation and Preliminaries

A *tree T *is a connected acyclic graph, consisting of a node set *V *(*T*) and an edge set *E*(*T*). *T *is *rooted *if it has exactly one distinguished node called the *root *which we denote by *rt*(*T*). Throughout this work, the term tree refers to a rooted tree. We define ≤_*T *_to be the partial order on *V *(*T*) where *x *≤_*T *_*y *if *y *is a node on the path between *rt*(*T*) and *x*. The set of minima under ≤_*T *_is denoted by ℒ(*T*) and its elements are called *leaves*. The set of all *non-root internal nodes *of *T*, denoted by *I*(*T*), is defined to be the set *V *(*T*)\(ℒ(*T*) ∪ {*rt*(*T*)}). If {*x, y*} ∈ *E*(*T*) and *x *≤_*T*_*y *then we call *y *the *parent *of *x *denoted by *pa*_*T *_(*x*) and we call *x *a *child *of *y*. The set of all children of *y *is denoted by *Ch*_*T*_(*y*). *T *is *fully binary *if every node has either zero or two children. If two nodes in *T *have the same parent, they are called *siblings*. The *least common ancestor *of a non-empty subset *L *⊆ *V *(*T*), denoted as *lca*(*L*), is the unique smallest upper bound of *L *under ≤_*T*_. The *subtree *of *T *rooted at node *y *∈ *V*(*T*), denoted by *T*_*y*_, is the tree induced by {*x *∈ *V *(*T*): *x *≤ *y*}. For each node *v *∈ *I*(*T*), the *cluster *(*v*) is defined to be the set of all leaf nodes in *T*_*v*_; i.e. (*v*) = ℒ(*T*_*v*_). We denote the set of all clusters of a tree *T *by ℋ(*T*). Given a set *L *⊆ ℒ(*T*), let *T' *be the minimal rooted subtree of *T *with leaf set *L*. We define the *leaf induced subtree T *[*L*] of *T *on leaf set *L *to be the tree obtained from *T' *by successively removing each non-root node of degree two and adjoining its two neighbors. The *symmetric difference *of two sets *A *and *B*, denoted by *A*Δ*B*, is the set (*A*\*B*) ∪ (*B*\*A*). A *profile * is a tuple of trees (*T*_1_, ..., *T*_*k*_).

### The RF Supertree Problem

Given a profile , we define a *supertree *on  to be a fully binary tree T* where .

**Definition 1 **(RF Distance). *Given a profile * = (*T*_1_, ..., *T*_*k*_) and a supertree T * *on *, *we define the *RF distance *as follows*:

*1. For any T*_*i*_, *where *1 ≤ *i *≤ *k*, *RF *(*T*_*i*_, T*) = |ℋ(*T*_*i*_)Δℋ(T*[ℒ(*T*_*i*_)])|.

*2*. 

*3. Let **be the set of supertrees on *, *then *.

*Remark*: Traditionally, the value of the RF distance, as computed above, is normalized by multiplying by 1/2. However, this does not affect the definition or computation of RF supertrees, and therefore, we do not normalize the RF distance.

**Problem 1 **(RF Supertree).

Instance: *A profile *.

Find: *A supertree T*_*opt*_*on **such that RF *(, *T*_*opt*_) = *RF *().

Recall that the RF Supertree problem is NP-hard [27].

### Local Search Problems

Here we first provide definitions for the re-rooting operation (denoted RR) and the TBR[22] and SPR[35] edit operations and then formulate the related local search problems that were motivated in the introduction.

**Definition 2 **(RR operation). *Let T be a tree and x *∈ *V *(*T*). RR(*T, x*) *is defined to be the tree T, if x *= *rt*(*T*) *or x *∈ *Ch*(*rt*(*T*))*. Otherwise*, RR(*T, x*) *is the tree that is obtained from T by (i) suppressing rt*(*T*), *and (ii) subdividing the edge *{*pa*(*x*), *x*} *by a new root node. We define the following extension*: RR(*T*) = ∪_*x *∈ *V*(*T*)_{RR(*T, x*)}.

For technical reasons, before we can define the TBR operation, we need the following definition.

**Definition 3 **(Planted tree). *Given a tree T, the *planted tree Φ(*T*) *is the tree obtained by adding a *root edge {*p, rt*(*T*)}, *where p *∉ *V *(*T*), *to T*.

**Figure 1:**
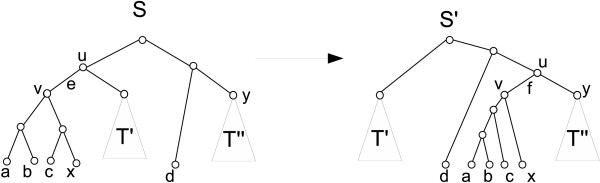
**TBR Operation**. Example depicting a TBR operation which transforms tree *S *into tree *S' *= TBR_*S*_(*v, x, y*).

**Definition 4 **(TBR operation). (*See Fig*. 1) *Let T be a tree, e *= (*u, v*) ∈ *E*(*T*), *where u *= *pa*(*v*), *and X, Y be the connected components that are obtained by removing edge e from T where v *∈ *X and u *∈ *Y. We define *TBR_*T*_(*v, x, y*) *for x *∈ *X and y *∈ *Y to be the tree that is obtained from *Φ(*T*) *by first removing edge e, then replacing the component X by *RR(*X, x*), *and then adjoining a new edge f between x' *= *rt*(RR(*X, x*)) *and Y as follows*:

*1. Create a new node y' that subdivides the edge *(*pa*(*y*), *y*).

*2. Adjoin the edge f between nodes x' and y'*.

*3. Suppress the node u, and rename x' as v and y' as u*.

*4. Contract the root edge*.

**Notation**. We define the following:

*1*. TBR_*T *_(*v, x*) = ∪_*y *∈ *Y *_{TBR_*T *_(*v, x, y*)}

*2*. TBR_*T *_(*v*) = ∪_*x *∈ *X *_TBR_*T *_(*v, x*)

*3*. TBR_*T *_= ∪_(*u*, *v*) ∈ *E*(*T*) _TBR_*T*_(*v*)

**Definition 5 **(SPR operation). *Let T be a tree, e *= (*u, v*) ∈ *E*(*T*), *where u *= *pa*(*v*), *and X, Y be the connected components that are obtained by removing edge e from T where v *∈ *X and u *∈ *Y. We define *SPR_*T *_(*v, y*), *for y *∈ *Y, to be the tree *TBR_*T *_(*v, v, y*)*. We say that the tree *SPR_*T *_(*v, y*) *is obtained from T by a *subtree prune and regraft (SPR) *operation that *prunes *subtree T*_*v *_*and *regrafts *it above node y*.

**Notation**. We define the following:

*1*. SPR_*T *_(*v*) = ∪_*y *∈ *Y*_{SPR_*T *_(*v, y*)}

*2*. SPR_*T *_= ∪_(*u*, *v*) ∈ *E*(*T*) _SPR_*T*_(*v*)

Note that an SPR operation for a given tree *T *can be briefly described through the following four steps: (i) prune some subtree *P *from *T*, (ii) add a root edge to the remaining tree *S*, (iii) regraft *P *into an edge of the remaining tree *S*, and (iv) contract the root edge.

We now define the relevant local search problems based on the TBR and SPR operations.

**Problem 2 **(TBR-Scoring (TBR-S)). *Given instance *⟨, *T*⟩, *where **is the profile *(*T*_1_, ..., *T*_*k*_) *and T is a supertree on *, find a tree T* ∈ TBR_*T *_*such that *.

**Problem 3 **(TBR-Restricted Scoring (TBR-RS)). *Given instance *⟨, *T*, *v*⟩, *where **is the profile *(*T*_1_, ..., *T*_*k*_), *T is a supertree on *, *and v is a non-root node in V *(*T*), find a tree T * ∈ TBR_*T *_(*v*) *such that *.

The problems SPR-*Scoring *(SPR-*S*) and SPR-*Restricted Scoring *(SPR-*RS*) are defined analogously to the problems TBR-S and TBR-RS respectively.

Throughout the remainder of this manuscript, *k *is the number of trees in the profile , *T *denotes a supertree on , and *n *is the number of leaves in *T*. The following observation follows from Definition 4.

**Observation 1**. *The *TBR-*S problem on instance *⟨, *T*⟩ *can be solved by solving the *TBR-*RS problem *|*E*(*T*)| *times*.

We show how to solve the TBR-S problem on the instance ⟨, *T*⟩ in *O*(*kn*^2^) time. Since SPR_*T *_⊆ TBR_*T *_this also implies an *O*(*kn*^2^) solution for the SPR-S problem. This gives a speed-up of Θ(*n*^2^) and Θ(*n*) over the best known (naïve) algorithms for the TBR-S and SPR-S problems respectively.

In particular, we first show that any instance of the TBR-RS problem can be decomposed into an instance of an SPR-RS problem, and an instance of a Rooting problem (defined in the next section). We show how to solve both these problems in *O*(*kn*) time, yielding an *O*(*kn*) time solution for the TBR-RS problem. This immediately implies an *O*(*kn*^2^) time algorithm for the TBR-S problem (see Observation 1).

Note that the size of the set TBR_*T *_is Θ(*n*^3^). Thus, for each tree in the input profile the time complexity of computing and enumerating the RF distances of all trees in TBR_*T *_is Ω(*n*^3^). However, to solve the TBR-S problem one only needs to find a tree with the minimum RF distance. This lets us solve the TBR-S problem in time that is sub-linear in the size of TBR_*T*_. In fact, after the initial *O*(*kn*^2^) preprocessing step, our algorithm can output the RF distance of any tree in TBR_*T *_in *O*(1) time.

## Structural Properties

Throughout this section, we limit our attention to one tree *S *from the profile . We show how to solve the TBR-RS problem for the instance ⟨(*S*), *T*, *v*⟩ for some non-root node *v *∈ *V *(*T*) in *O*(*n*) time. Based on this solution, it is straightforward to solve the TBR-RS problem on the instance ⟨, *T*, *v*⟩ with-in *O*(*kn*) time as well. For clarity, we will also assume that ℒ(*S*) = ℒ(*T*). In general, if ℒ(*S*) ⊂ ℒ(*T*) then we can simply set *T *to be *T *[ℒ(*S*)]. This takes *O*(*n*) time and, consequently, does not affect the time complexity of our algorithm.

Our algorithm makes use of the LCA mapping from *S *to *T*. This mapping is defined as follows.

**Definition 6 **(LCA Mapping). *Given two trees T' *and T such that ℒ(*T'*) ⊆ ℒ(*T*), *the *LCA mapping ℳ_*T'*, *T*_: *V*(*T'*) → *V*(*T*) *is the mapping *ℳ_*T'*, *T *_(*u*) = *lca*_*T *_(ℒ()).

**Notation**. We define a boolean function *f*_*T*_: *I*(*S*) → {0, 1} such that *f*_*T *_(*u*) = 1 if there exists a node *v *∈ *I*(*T*) such that (*u*) = (*v*), and *f*_*T *_(*u*) = 0 otherwise. Thus, *f*_*T *_(*u*) = 1 if and only if the cluster (*u*) exists in the tree *T *as well. Additionally, we define ℱ_*T *_= {*u *∈ *I*(*S*): *f*_*T *_(*u*) = 0}; that is, ℱ_*T *_is the set of all nodes *u *∈ *I*(*S*) such that the cluster (*u*) does not exist in the tree *T*.

The following lemma associates the value *RF*(*S, T*) with the cardinality of the set ℱ_*T*_.

**Lemma 1**. *RF*(*S, T*) = |*I*(*T*)| - |*I*(*S*)| + 2·|ℱ_*T *_|.

*Proof*. Let  denote the set {*u *∈ *I*(*S*): *f*_*T *_(*u*) = 1}. By the definition of *RF *(*S, T*), we must have *RF*(*S, T*) = |*I*(*T*)| + |*I*(*S*)| - 2·||. And hence, since || + |ℱ_*T *_| = *I*(*S*), we get *RF*(*S, T*) = |*I*(*T*)| - |*I*(*S*)| + 2·|ℱ_*T *_|.    □

**Lemma 2**. *For any u *∈ *I*(*S*), *f*_*T *_(*u*) = 1 *if and only if *|(*u*)| = |(ℳ_*S*, *T *_(*u*))|.

*Proof*. If |(*u*)| = |(ℳ_*S*, *T*_(*u*))| then we must have (*u*) = (ℳ_*S*, *T *_(*u*)) and, consequently, *f*_*T *_(*u*) = 1. In the other direction, if |(*u*)| ≠ |(ℳ_*S*, *T *_(*u*))|, then we must have (*u*) ⊂ (ℳ_*S*, *T *_(*u*)) and, consequently, *f*_*T *_(*u*) = 0.    □

The LCA mapping from *S *to *T *can be computed in *O*(*n*) time [39], and consequently, by Lemmas 1 and 2, we can compute the RF distance between *S *and *T *in *O*(*n*) time as well (other *O*(*n*)-time algorithms for calculating the RF distance are presented in [30,31]). Moreover, Lemma 1 implies that in order to find a tree T* ∈ TBR_*T *_(v) such that , it is sufficient to find a tree T* ∈ TBR_*T *_(*v*) for which .

*Remark*: An implicit assumption here is that the leaves of both trees are labeled by integers {1, ..., *n*}. If the leaf labels are arbitrary, then we require an additional *O*(*kn *log *n*)-time preprocessing step to relabel the leaves of the trees in the given profile. Note, however, that this additional step does not add to the overall time complexity of solving the TBR-S or SPR-S problems.

We now show that the TBR-RS problem can be solved by solving two smaller problems separately and combining their solutions.

As before, we limit our attention to one tree *S *from the profile . Given the TBR-RS instance ⟨(*S*), *T*, *v*⟩, we define a bipartition {*X*, } of *I*(*S*), where *X *= {*u *∈ *I*(*S*): ℳ_*S*, *T*_(*u*) ∈ *V *(*T*_*v*_)}.

**Lemma 3**. *If u *∈ *X, then f*_*T'*_(*u*) = *f*_*T *_(*u*) *for all T *' ∈ TBR_*T *_(*v, v*). *If u *∈ *and y denotes the sibling of v, then f*_*T'*_(*u*) = *f*_*T *_(*u*), *where T' *= TBR_*T*_(*v*, *x*, *y*) *for any x *∈ *V *(*T*_*v*_).

*Proof*. Consider the case when *u *∈ *X*. Let *T' *be any tree in TBR_*T*_(*v*, *v*) and let node *y *∈ *V *(*T*) be such that *T' *= TBR(*v*, *v*, *y*). Thus, for any node *w *∈ *V *(*T*_*v*_), the subtrees *T*_*v *_and  must be identical. Since *u *∈ *X*, we must have ℳ_*S*, *T *_(*u*) ∈ *T*_*v *_and, consequently, . Lemma 2 now implies that *f*_*T' *_(*u*) = *f*_*T *_(*u*).

Now consider the case when *u ∈ *. Node *y *denotes the sibling of *v *in tree *T *and let *T' *= TBR(*v*, *x*, *y*), for some *x *∈ *V *(*T*_*v*_). Thus, for any node *w *∈ *V*(*T*)\*V*(*T*_*v*_), we must have ℒ_*T*_(*w*) = ℒ_*T' *_(*w*). Moreover, the leaf sets of the two subtrees rooted at the children of *w *in *T *must be identical to the leaf sets of the two subtrees rooted at the children of *w *in *T'*: This implies that if ℳ_*S*, *T*_(*u*) = *w*, then ℳ_*S*, *T' *_(*u*) = *w *as well. By Lemma 2 we must therefore have *f*_*T' *_(*u*) = *f*_*T *_(*u*).    □

Lemma 3 implies that a tree in TBR_*T *_(*v*) with smallest RF distance can be obtained by optimizing the rooting for the pruned subtree, and optimizing the regraft location separately. This allows us to obtain a tree in TBR_*T *_(*v*) with smallest RF distance by evaluating only *O*(*n*) trees. Contrast this with the naïve approach to finding a tree in TBR_*T *_(*v*) with smallest total distance, which is to evaluate all trees obtained by rerooting the pruned subtree in all possible ways, and, for each rerooting, regrafting the subtree in all possible locations. Since there are *O*(*n*) ways to reroot the pruned subtree, and *O*(*n*) ways to regraft, this would require evaluating *O*(*n*^2^) trees. It is interesting to note that this ability to decompose the TBR-RS problem into two simpler problems is not unique to the context of RF supertrees alone. For example, it has been observed that a similar decomposition can be achieved in the context of the gene duplication problem [37].

Thus, to solve the TBR-RS problem, we must find (i) a rerooting *T' *of the subtree *T*_*v *_for which ℱ_*T' *_is minimized, and (ii) a regraft location *y *for *T*_*v *_which minimizes |ℱ_SPR__(*v*, *y*)_|. Observe that the problem in part (ii) is simply the SPR-RS problem on the input instance ⟨(*S*), *T*, *v*⟩. For part (i), consider the following problem statement.

**Problem 4 **(Rooting). *Given instance *⟨, *T*, *v*⟩, *where **is the profile *(*T*_1_, ..., *T*_*k*_), *T is a supertree on *, *and v is a non-root node in V *(*T*), *find a node x *∈ *V *(*T*_*v*_) *for which RF *(, TBR_*T *_(*v, x, y*)) *is minimum, where y denotes the sibling of v in T*.

Note that the problem in part (i) is the Rooting problem on the input instance ⟨(*S*), *T*, *v*⟩. We show how to solve both the Rooting and the SPR-RS problems in *O*(*n*) time on instance ⟨(*S*), *T*, *v*⟩. As seen above, based on Lemma 3, this immediately implies that the TBR-RS problem for a profile consisting of a single tree can be solved in *O*(*n*) time. To solve the TBR-RS problem on instance ⟨, *T*, *v*⟩, we simply solve the Rooting and SPR-RS problems separately on the input instance ⟨, *T*, *v*⟩, which takes *O*(*kn*) time (see Theorems 3 and 4). We thus have the following two theorems.

**Theorem 1**. *The *TBR-*RS problem can be solved in O*(*kn*) *time*.

**Theorem 2**. *The *TBR-*S problem can be solved in O*(*kn*^2^) *time*.

## Solving the Rooting Problem

To solve the Rooting problem on instance ⟨(*S*), *T*, *v*⟩, we rely on an efficient algorithm for computing the value of *f*_*T' *_(*u*) for any *T' *∈ RR(*T*_*v*_) and any *u *∈ *I*(*S*). This algorithm relies on the following five lemmas. Let *a *denote the node ℳ_*S*, *T *_(*u*), *y *denote the sibling of *v *in *T*, and *T' *= TBR_*T*_(*v, x, y*) for *x *∈ *V *(*T*_*v*_). Depending on *a *and *f*_*T *_(*u*) there are five possible cases: (i) *a *∉ *V *(*T*_*v*_), (ii) *a *= *rt*(*T*_*v*_) and *f*_*T *_(*u*) = 1, (iii) *a *= *rt*(*T*_*v*_) and *f*_*T *_(*u*) = 0, (iv) *a *∈ *V *(*T*_*v*_)\*rt*(*T*_*v*_) and *f*_*T *_(*u*) = 1, and (v) *a *∈ *V *(*T*_*v*_)\*rt*(*T*_*v*_) and *f*_*T *_(*u*) = 0. Lemmas 4 through 8 characterize the value *f*_*T' *_(*u*) for each of these five cases respectively.

**Lemma 4**. *If a *∉ *V *(*T*_*v*_), *then f*_*T' *_(*u*) = *f*_*T *_(*u*) *for any x *∈ *V *(*T*_*v*_).

*Proof*. Follows directly from Lemma 3.    □

**Lemma 5**. *If a *= *rt*(*T*_*v*_) *and f*_*T *_(*u*) = 1, *then f*_*T' *_(*u*) = 1 *for all x *∈ *V *(*T*_*v*_).

*Proof*. Since we have *a *= *rt*(*T*_*v*_) and *f*_*T *_(*u*) = 1, by Lemma 2 we must have ℒ(*S*_*u*_) = ℒ(*T*_*v*_). Thus, for any *x *∈ *V *(*T*_*v*_), ℳ_*S*, *T' *_(*u*) must be the root of the subtree RR(*T*_*v*_, *x*). The lemma follows.    □

**Lemma 6**. *Let L denote the set *ℒ(*T*_*v*_)\ℒ(*S*_*u*_), *and let * (*L*)*. If a *= *rt*(*T*_*v*_) *and f*_*T *_(*u*) = 0, *then*,

*1. for **b, f*_*T' *_(*u*) = 0, *and*,

*2. for **b, f*_*T' *_(*u*) = 1 *if and only if *|*L*| = |ℒ(*T*_*b*_)|.

*Proof*. Since *a *= *rt*(*T*_*v*_) and *f*_*T *_(*u*) = 0, by Lemma 2 we must have ℒ(*S*_*u*_) ≠ ℒ(*T*_*v*_). We analyze each part of the lemma separately.

1. *b*: For this case to be valid, we must have *rt*(*T*_*v*_). Therefore, let  (*b*). For any *T' *in this case, *b' *= *pa*_*T' *_(*b*). Moreover, ℒ() ∩ ℒ(*S*_*u*_) ≠ ∅. Therefore, we must have *b *<_*T' *_ℳ_*S*, *T' *_(*u*) Hence,  in this case, and, consequently, Lemma 2 implies that *f*_*T' *_(*u*) = 0.

2. *b*: We divide our analysis into two cases:

(a) |*L*| = |ℒ(*T*_*b*_)|: In this case we must have *b *≠ *rt*(*T*_*v*_). Therefore, let *b' *denote the parent of *b *in tree *T*_*v*_. Now consider the tree *T'*. The set ℒ() must be identical to ℒ(*S*_*u*_). Hence, *f*_*T' *_(*u*) = 1 in this case.

(b) |*L*| ≠ |ℒ(*T*_*b*_)|: We claim that there does not exist any edge (*pa*(*w*), *w*) ∈ *E*(*T*_*v*_) such that ℒ(*T*_*w*_) is either ℒ(*S*_*u*_) or *L*. Let us suppose, for the sake of contradiction, that such an edge exists. If ℒ(*T*_*w*_) = ℒ(*S*_*u*_) then we must have *a *= *w*, which is a contradiction since *a *= *rt*(*T*_*v*_). If ℒ(*T*_*w*_) = g *L *then we must have *b *= *w*, and, consequently, |*L*| ≠ |ℒ(*T*_*b*_)|, which is, again, a contradiction. Thus, such an edge (*pa*(*w*), *w*) ∈ *E*(*T*_*v*_) cannot exist. Hence, we must have *f*_*T' *_(*u*) = 0 for every *x *∈ *V *(*T*_*v*_) in this case.

The lemma follows.    □

**Lemma 7**. *If a *∈ *V *(*T*_*v*_)\*rt*(*T*_*v*_) *and f*_*T*_(*u*) = 1, *then f*_*T' *_(*u*) = 0 *if and only if x < T*_*v *_*a*.

*Proof*. By Lemma 2 we must have ℒ(*S*_*u*_) = ℒ(*T*_*a*_). We have two cases:

1. *a*: In this case we must have ℳ_*S*, *T' *_(*u*) = *a*, and ℒ(*T*_*a*_) = ℒ(). Thus, ℒ(*S*_*u*_) = ℒ() and hence, *f*_*T' *_(*u*) = 1.

2. *a*: In this case, ℳ_*S*, *T' *_(*u*) must be the root of the subtree RR(*T*_*v*_, *x*). Since ℒ(RR(*T*_*v*_, *x*)) = ℒ(*T*_*v*_), and ℒ(*S*_*u*_) ≠ ℒ(*T*_*v*_), Lemma 2 implies that *f*_*T' *_(*u*) = 0.

The lemma follows.    □

**Lemma 8**. *If a *∈ *V *(*T*_*v*_)\*rt*(*T*_*v*_) *and f*_*T *_(*u*) = 0, *then f*_*T' *_(*u*) = 0 *for all x *∈ *V *(*T*_*v*_).

*Proof*. By Lemma 2 we must have ℒ(*S*_*u*_) ≠ ℒ(*T*_*a*_). We have two possible cases:

1. *a*: In this case we must have ℳ_*S*, *T' *_(*u*) = *a*, and ℒ(*T*_*a*_) = ℒ(). Thus, ℒ(*S*_*u*_) ≠ ℒ() and hence, *f*_*T' *_(*u*) = 0

2. *a*: In this case, ℳ_*S*, *T' *_(*u*) must be the root of the subtree RR(*T*_*v*_, *x*). Since ℒ(RR(*T*_*v*_, *x*)) = ℒ(*T*_*v*_), and ℒ(*S*_*u*_) ≠ ℒ(*T*_*v*_), Lemma 2 implies that *f*_*T' *_(*u*) = 0.

The lemma follows.

**The Algorithm**. For any *x *∈ *V *(*T*_*v*_) let *A*(*x*) denote the cardinality of the set

{*u *∈ *I*(*S*): *f*_*T *_(*u*) = 0, but *f*_*T' *_(*u*) = 1}, and *B*(*x*) the cardinality of the set

{*u *∈ *I*(*S*): *f*_*T *_(*u*) = 1, but *f*_*T' *_(*u*) = 0}, where *T' *= TBR_*T *_(*v, x, y*).

By definition, to solve the Rooting problem we must find a node *x *∈ *V *(*T*_*v*_) for which |*A*(*x*)| - |*B*(*x*)| is maximized. Our algorithm computes, at each node *x *∈ *V *(*T*_*v*_), the values *A*(*x*) and *B*(*x*).

In a preprocessing step, our algorithm computes the mapping ℳ_*S*, *T *_as well as the size of each cluster in *S *and *T*, and creates and initializes (to 0) two counters *α*(*x*) and *β*(*x*) at each node *x *∈ *V *(*T*_*v*_). This takes *O*(*n*) time. When the algorithm terminates, the values *α*(*x*) and *β*(*x*) at any *x *∈ *V *(*T*_*v*_) will be the values *α*(*x*) and *β*(*x*).

Recall that, given *u *∈ *I*(*S*), *a *denotes the node ℳ_*S*, *T *_(*u*). Thus, any given *u *∈ *I*(*S*) must satisfy the precondition (given in terms of *a*) of exactly one of the the Lemmas 4 through 8. Moreover, the precondition of each of these lemmas can be checked in *O*(1) time.

The algorithm then traverses through *S *and considers each node *u *∈ *I*(*S*). There are three cases:

1. If *u *satisfies the preconditions of Lemmas 4, 5, or 8 then we must have *f*_*T' *_(*u*) = *f*_*T *_(*u*). Consequently, we do nothing in this case.

2. If *u *satisfies the precondition of Lemma 7, then we increment the value of *β*(*x*) at each node *x *∈ *V *(*T*_*a*_)\{*a*} (where *a *is as in the statement of Lemma 7). To do this efficiently we can simply increment a counter at node *a *such that, after all *u *∈ *I*(*S*) have been considered, a single pre-order traversal of *T*_*v *_can be used to compute the correct values of *β*(*x*) at each *x *∈ *V *(*T*_*v*_).

3. If *u *satisfies the precondition of Lemma 6, then we proceed as follows: Let *a *and *L *be as in the statement of Lemma 6. According to the Lemma, if we can find a node *b *∈ *V *(*T*_*v*_) such that (L) and |ℒ(*T*_*b*_)| = |*L*|, then we increment the value of *α*(*x*) at each node *x *∈ *V *(*T*_*b*_); otherwise, if such a *b *does not exist, we do nothing. As before, to do this efficiently, we only increment a single counter at node *b *such that, after all *u *∈ *I*(*S*) have been considered, a pre-order traversal of *T*_*v *_suffices to compute the correct values of *α*(*x*) at each *x *∈ *V *(*T*_*v*_). In order to prove the *O*(*n*) run-time for this algorithm we will now explain how to precompute such a corresponding node *b *(if it exists), for each *u *∈ *I*(*S*) satisfying the precondition of Lemma 6, within *O*(*n*) time. Note that any edge in a tree bi-partitions its leaf set. Construct the tree *S' *= *S *[ℒ(*T*_*v*_)]. Observe that, given any candidate *u*, the corresponding node *b *exists if and only if the partition of ℒ(*S'*) induced by the edge (*u, pa*(*u*)) *E*(*S'*), is also induced by some edge, *e*, in the tree *T*_*v *_If such an *e *exists, then *b *must be that node on *e *which is farther away from the root, i.e. the edge *e *must be the edge (*b, pa*(*b*)) in *T*_*v *_This edge *e *(or its absence) can be precomputed, for all candidate *u*, as follows: Compute the strict consensus of the unrooted variants of the trees *S' *and *T*_*v*_. Every edge in this strict consensus corresponds to an edge in *S' *and an edge in *T*_*v *_that induce the same bi-partitions in the two trees.

Thus, for all candidate *u *that lie on such an edge, the corresponding node *b *can be inferred in *O*(1) time (by using the association between the edges of the strict consensus and the edges of *S' *and *T*_*v*_), and for all candidate *u *that do not lie on such an edge, we know that the corresponding node *b *does not exist. This strict consensus of the unrooted variants of *S' *and *T*_*v *_can be precomputed with-in *O*(*n*) time by using the algorithm of Day [30].

Hence, the Rooting problem for a profile consisting of a single tree can be solved in *O*(*n*) time; yielding the following theorem.

**Theorem 3**. *The *Rooting *problem can be solved in O*(*kn*) *time*.

## Solving the SPR-RS Problem

We will show how to solve the SPR-RS problem on instance ⟨(*S*), *T, v*⟩ in *O*(*n*) time. Consider the tree *R *= SPR_*T *_(*v, rt*(*T*)) Observe that, since SPR_*R*_(*v*) = SPR_*S*_(*v*), solving the SPR-RS problem on instance ⟨(*S*), *T, v*⟩ is equivalent to solving it on the instance ⟨(*S*), *R, v*⟩. Thus, in the remainder of this section, we will work with tree *R *instead of tree *S*. The following four lemmas let us efficiently infer, for any *u *∈ *I*(*S*), whether *f*_*T' *_(*u*) = 1 or *f*_*T' *_(*u*) = 0, for any given *T' *∈ SPR_*R*_(*v*).

For brevity, let *a *denote the node ℳ_*S*, *R*_(*u*), and let *Q *denote the set *V *(*R*)\(*V *(*R*_*v*_) ∪ {*rt*(*R*)}). Let *T' *= SPR_*R*_(*v, x*), for any *x *∈ *Q*.

Depending on *a *and *f*_*R*_(*u*) there are four possible cases: (i) *a *∈ *V *(*R*_*v*_), (ii) *a *∈ *Q *and *f*_*R*_(*u*) = 1, (iii) *a *∈ *Q *and *f*_*R*_(*u*) = 0, and (iv) *a *= *rt*(*R*). Lemmas 9 through 12 characterize the value *f*_*T' *_(*u*) for each of these four cases respectively.    □

**Lemma 9**. *If a *∈ *V *(*R*_*v*_), *then f*_*T' *_(*u*) = *f*_*R*_(*u*) *for any x *∈ *Q*.

*Proof*. Observe that TBR_*R*_(*v, v*) = SPR_*R*_(*v*). Lemma 3 now immediately completes the proof.    □

**Lemma 10**. *If a *∈ *Q and f*_*R*_(*u*) = 1, *then*,

*1. f*_*T' *_(*u*) = 0, *for x *<_*R *_*a, and*

*2. f*_*T' *_(*u*) = 1, *otherwise*.

*Proof*. Since *f*_*R*_(*u*) = 1, Lemma 2 implies that |(*u*)| = |(*a*)|. Let *T' *= SPR_*R*_(*v, x*); we now have two cases.

1. *x *<_*R *_*a*: In this case ℳ_*S*, *T' *_(*u*) = *a*, and, since |(*u*)| < |(*a*)| < |(*a*)|, we must have *f*_*T' *_(*u*) = 0 (by Lemma 2).

2. *x *≮_*R *_*a*: In this case ℳ_*S*, *T' *_(*u*) = *a*, and since |(*u*)| = |(*a*)| = |(*a*)|, we must have *f*_*T' *_(*u*) = 1 (by Lemma 2).

The lemma follows.    □

**Lemma 11**. *If a *∈ *Q and f*_*R*_(*u*) = 0, *then f*_*T' *_(*u*) = 0 *for any x *∈ *Q*.

*Proof*. Since *f*_*R*_(*u*) = 0, Lemma 2 implies that |(*u*)| ≠ |(*a*)|. Thus, by the definition of LCA mapping, |(*u*)| < |(*a*)|. Let *T' *= SPR_*R*_(*v, x*); we now have two cases.

1. *x *<_*R *_*a*: In this case ℳ_*S*, *T' *_(*u*) = *a*, and, since |(*u*)| < |(*a*)| < |(*a*)|, we must have *f*_*T' *_(*u*) = 0 (by Lemma 2).

2. *x *≮_*R *_*a*: In this case ℳ_*S*, *T' *_(*u*) = *a*, and since |(*u*)| < |(*a*)| = |(*a*)|, we must have *f*_*T' *_(*u*) = 0 (by Lemma 2).

The lemma follows.    □

For the next lemma, let *S' *be the tree obtained from *S *by suppressing all nodes *s *for which ℳ_*S*, *R*_(*s*) ∈ *R*_*v*_.

**Lemma 12**. *If a *= *rt*(*R*) *and b *= ℳ_*S'*, *R*_(*u*), *then, f*_*T' *_(*u*) = 1 *if and only if x *<_*R *_*b and *|ℒ(*R*_*b*_)| + |ℒ(*R*_*v*_)| = |ℒ(*S*_*u*_)|.

*Proof*. First, observe that, since *a *= *rt*(*R*), the mapping ℳ_*S'*, *R*_(*u*) is well defined. Second, since *b *= ℳ_*S'*, *R*_(*u*), we must have ℒ() ⊆ ℒ(*R*_*b*_), which implies that ℒ(*S*_*u*_) ⊆ ℒ(*R*_*v*_) ⊆ ℒ(*R*_*b*_). We now have the following three cases:

1. *x *≮_*R *_*b*: In this case we must have ℳ_*S*, *T' *_(*u*) = *lca*_*T' *_(*x*, *b*). By Lemma 2 we know that *f*_*T' *_(*u*) = 1 only if |(*u*)| = |(ℳ_*S*, *T' *_(*u*))|. However, since we have ℒ(*S*_*u*_) ⊆ ℒ(*R*_*v*_) ⊆ ℒ(*R*_*b*_), and *x *≮_*R *_*b*, we must have |(*u*)| < |(ℳ_*S*, *T' *_(*u*))|; and hence, *f*_*T' *_(*u*) = 0.

2. *x *<_*R *_*b ***and **|ℒ(*R*_*b*_)| + |ℒ(*R*_*v*_)| ≠ |ℒ(*S*_*u*_)|: In this case we must have ℳ_*S*, *T' *_(*u*) = *b*. Since |ℒ(*R*_*b*_)| + |ℒ(*R*_*v*_)| ≠ |ℒ(*S*_*u*_)|, we must have ℒ(*S*_*u*_) ⊂ ℒ(*R*_*v*_) ∪ ℒ(*R*_*b*_), which implies that |(*u*)| < |(ℳ_*S*, *T' *_(*u*))|. Thus, by Lemma 2, we must have *f*_*T' *_(*u*) = 0.

3. *x *<_*R *_*b ***and **|ℒ(*R*_*b*_)| + |ℒ(*R*_*v*_)| = |ℒ(*S*_*u*_)|: In this case we must have ℳ_*S*, *T' *_(*u*) = *b*. Moreover, since |ℒ(*R*_*b*_)| + |ℒ(*R*_*v*_)| = |ℒ(*S*_*u*_)|, we must have |(*u*)| = |(ℳ_*S*, *T' *_(*u*))|. Thus, by Lemma 2, we must have *f*_*T' *_(*u*) = 1.

The lemma follows.    □

**The Algorithm**. Note that SPR_*T *_(*v*) = SPR_*R*_(*v*) = ∪_*x *∈ *Q *_SPR_*R*_(*v, x*). For any *x *∈ *Q*, let *A*(*x*) = |{*u *∈ *I*(*S*): *f*_*R*_(*u*) = 0, but *f*_*T' *_(*u*) = 1}|, and *B*(*x*) = |{*u *∈ *I*(*S*): *f*_*R*_(*u*) = 1, but *f*_*T' *_(*u*) = 0}|, where *T' *= SPR_*R*_(*v, x*). By definition, to solve the SPR-RS problem on instance ⟨(*S*), *T, v*⟩ we must find a node *x *∈ *Q *for which |*A*(*x*)| - |*B*(*x*)| is maximized. Our algorithm computes, at each node *x *∈ *Q*, the values *A*(*x*) and *B*(*x*).

In a preprocessing step, our algorithm first constructs the tree *R *computes the mapping ℳ_*S*, *R *_as well as the size of each cluster in *S *and *R*, and creates and initializes (to 0) two counters *α*(*x*) and *β*(*x*) at each node *x *∈ *Q*. This takes a total of *O*(*n*) time. When the algorithm terminates, the values *α*(*x*) and *β*(*x*), at any *x *∈ *Q *will be the values *A*(*x*) and *B*(*x*).

Recall that, given *u *∈ *I*(*S*), *a *denotes the node ℳ_*S*, *R*_(*u*). Thus, any given *u *∈ *I*(*S*) must satisfy the precondition (given in terms of *a*) of exactly one of the the Lemmas 9 through 12. Moreover, the precondition of each of these lemmas can be checked in *O*(1) time.

The algorithm then traverses through *S *and considers each node *u *∈ *I*(*S*). There are three cases:

1. If *u *satisfies the preconditions of Lemmas 9 or 11 then we must have *f*_*T' *_(*u*) = *f*_*R*_(*u*) Consequently, we do nothing in this case.

2. If *u *satisfies the precondition of Lemma 10, then we increment the value of *β*(*x*) at each node *x *∈ *V *(*T*_*a*_)\{*a*} (where *a *is as in the statement of Lemma 10). This can be done efficiently as shown in part (2) of the algorithm for the Rooting problem.

3. If *u *satisfies the precondition of Lemma 6, and if |ℒ(*R*_*b*_)| + |ℒ(*R*_*v*_)| = |ℒ(*S*_*u*_)|, then we increment the value of *α*(*x*) at each node *x *∈ *V *(*T*_*b*_)\{*b*} (where *a *and *b *are as in the statement of Lemma 6).

Again, to do this efficiently, we increment a counter at node *b*, and perform a subsequent pre-order traversal. Note also that the mapping ℳ_*S'*, *R *_can be computed in *O*(*n*) time in the preprocessing step, and hence the node *b *can be inferred in *O*(1) time. The condition |ℒ(*R*_*b*_)| + |ℒ(*R*_*v*_)| = |ℒ(*S*_*u*_)| is also verifiable in *O*(1) time.

Hence, the SPR-RS problem for a profile consisting of a single tree can be solved in *O*(*n*) time; yielding the following theorem.

**Theorem 4**. *The *SPR-*RS problem can be solved in O*(*kn*) *time*.

**Remark**. To improve the performance of local search heuristics in phylogeny construction, the starting tree for the first local search step is often constructed using a greedy 'stepwise addition' procedure. This greedy procedure builds a starting species tree step-by-step by adding one taxon at a time at its locally optimal position. In the context of RF supertrees, our algorithm for the SPR-RS problem also yields a Θ(*n*) speed-up over naïve algorithms for this greedy procedure.

## Experimental Evaluation

In order to evaluate the performance of the RF supertree method, we implemented an RF heuristic based on the SPR local search algorithm. We focused on the SPR local search because it is faster and simpler to implement than TBR, and in analyses of MRF and triplet supertrees, the performance of SPR and TBR was very similar [22,38]. We compared the performance of the RF supertree heuristic to MRP and the triplet supertree method (which seeks a supertree with the most shared triplets with the collection of input trees) using published supertree data sets from sea birds [40], marsupials [41], placental mammals [42], and legumes [43]. The published data sets contain between 7 and 726 input trees and between 112 and 571 total taxa (Table 1).

**Table 1:** Experimental Results.

Data Set	Supertree Method	RF-Distance	Parsimony Score
Marsupial (272 taxa; 158 trees)	RF-Ratchet	1514	2528
	RF-MRP	**1502**	2513
	MRP-TBR	1514	**2509**
	MRP-Ratchet	1514	**2509**
	Triplet	1604	2569

Sea Birds (121 taxa; 7 trees)	RF-Ratchet	**61**	223
	RF-MRP	**61**	223
	MRP-TBR	63	**221**
	MRP-Ratchet	63	**221**
	Triplet	**61**	223

Placental Mammals (116 taxa; 726 trees)	RF-Ratchet	**5686**	8926
	RF-MRP	5690	8890
	MRP-TBR	5694	**8878**
	MRP-Ratchet	5694	**8878**
	Triplet	6032	9064

Legumes (571 taxa; 22 trees)	RF-Ratchet	1556	965
	RF-MRP	**1534**	882
	MRP-TBR	1554	856
	MRP-Ratchet	1552	**854**
	Triplet	N/A	N/A

Experimental results comparing the performance of the RF supertree method to MRP and triplet supertree methods. We used five different supertree analyses: RF supertrees using our SPR local search algorithm with a ratchet starting from either random addition sequence trees (RF-ratchet) or MRP trees (RF-MRP), MRP with TBR branch swapping with (MRP-ratchet) and without (MRP-TBR) a ratchet search, and triplet supertrees with a TBR local search (Triplet). We measured the RF distance to the collection of input trees (RF-distance) and the parsimony score of a best found supertree based on the matrix representation of the input trees. The best RF distance and parsimony scores are in bold

There are a number of ways to implement any local search algorithm. Preliminary analyses of the RF heuristic based on the SPR local search indicated that, as with other phylogenetic methods, the starting tree can affect the estimate of the final supertree. Occasionally the SPR searches got caught in local optima with relatively high RF-distance scores. To ameliorate this potential problem, we implemented a ratchet search heuristic for RF supertrees based on the parsimony ratchet [44]. In general, a ratchet search performs a number of iterations -- in our case 25 -- that consist of two local SPR searches: one in which the characters (input trees) are equally weighted, and another in which the set of the characters are re-weighted. We re-weighted the characters by randomly removing approximately two-thirds of the input trees. The goal of re-weighting the characters is to alter the tree space to avoid getting caught in a globally suboptimal part of the tree space. At the end of each iteration, the best tree is taken as the starting point of the next iteration. For each data set, we started RF ratchet searches from 20 random sequence addition starting trees, and we also ran three replicates starting from an optimal MRP supertree. All RF supertree analyses were performed on an 3 GHz Intel Pentium 4 based desktop computer with 1 GB of main memory. The RF-ratchet runs took between 5 seconds (for the Sea Birds data set) and 90 minutes (for the legume dataset) when starting from a random sequence addition tree. RF-ratchet runs starting from optimal MRP trees were at least twice as fast because they required fewer search steps.

For our MRP analyses, we also tried two heuristic search methods, both implemented using PAUP* [18]. First, we performed 20 replicates of TBR branch swapping from trees built with random addition sequence starting trees. Next, we performed 20 replicates of a parsimony ratchet search with TBR branch swapping. Based on the results of trial analyses, each ratchet search consisted of 25 iterations, each reweighting 15% of the characters. The PAUP* command block for the parsimony ratchet searches was generated using PAUPRat [45]. For each data set, we performed 20 replicates of a TBR local search heuristic starting with random addition sequence trees. Triplet supertrees were constructed using the program from Lin et al. [38]. We were unable to perform ratchet searches with the existing triplet supertree software, and also, due to memory limitations, we were unable to perform triplet supertree analyses on the legume data set.

Our analyses demonstrate the effectiveness of our local search heuristics for the RF supertree problem. In all four data sets, RF-ratchet searches found the supertrees with the lowest total RF distance to the input trees (Table 1). MRP also generally performs well, finding supertrees with RF distances between 0.14% (placental mammals) and 3.3% (sea birds) higher than the best score found by the RF supertree heuristics (Table 1). The triplet supertree method performs as well as the RF supertree method on the small sea bird data set; however, the triplet supertrees for the marsupial and placental mammal data sets have a much higher RF distance to the input trees than either the RF or MRP supertrees (Table 1). For all the data sets, the MRP supertrees had the lowest (best) parsimony score based on a binary matrix representation of the input trees (Table 1). Thus, not surprisingly, it appears that optimizing based on the parsimony score or the triplet distance to the input trees does not optimize the similarity of the supertrees to the input trees based on the RF distance metric (see also [11,13]).

All of the data sets used in this analysis are from published studies that used MRP. Therefore, it is not surprising that MRP performed well (but see [46]). Still, our results demonstrate that MRP leaves some room for improvement. If the goal is to find the supertrees that are most similar to the collection of input trees, the RF searches ultimately provide better estimates than MRP (Table 1).

Interestingly, while the MRP trees tend to have relatively low RF-distance scores, in some cases, such as the legume data set, trees with low RF-distance scores have high parsimony scores (Table 1). Thus, parsimony scores are not necessarily indicative of RF score, and MRP and RF supertree optimality criteria are certainly not equivalent. Still, MRP trees appear to be useful as starting points for RF supertree heuristics. Indeed, in three of the four data sets, the best RF trees were found in ratchet searches beginning from MRP trees (Table 1).

Our program for computing RF supertrees is freely available (for Windows, Linux, and Mac OS X) at http://genome.cs.iastate.edu/CBL/RFsupertrees

## Discussion and Conclusion

There is a growing interest in using supertrees for large-scale evolutionary and ecological analyses. Yet there are many concerns about the performance of existing supertree methods, and the great majority of published supertree analyses have relied on only MRP [47]. Since the goal of a supertree analysis is to synthesize the phylogenetic data from a collection of input trees, it makes sense that an effective supertree method should directly seek the supertree that is most similar to the input trees. Our new algorithms make it possible, for the first time, to estimate large supertrees by directly optimizing the RF distance from the supertree to the input trees.

There are numerous alternate metrics to compare phylogenetic trees besides the RF distance, and any of these can be used for supertree methods (see, for example, [11]). Triplet distance supertrees [11,48], quartet-fit and quartet joining supertrees [11,24], maximum splits-fit supertrees [11], and most similar supertrees [49] are all, like RF supertrees, estimated by comparing input trees to the supertree using tree distance measures. All of these methods may provide different, and perhaps equally valid, perspectives on supertree accuracy. Based on our experimental analyses using the RF and triplet supertree method, optimizing the supertree based on different distance measures can result in very different supertrees (Table 1). In the future, it will be important to characterize and compare the performance of these methods in more detail (see, for example, [11,50]).

The results also suggest several future directions for research. Although heuristics guided by local search problems, especially SPR and TBR, have been very effective for many intrinsically difficult phylogenetic inference problems, our experiments indicate that the tree space for RF supertrees is complex. The ratchet approach and also starting from MRP trees appears to improve the performance in the four examples we tested (Table 1). However, more work is needed to identify the most efficient ways to implement our fast local search heuristics. Also, the use of alternative supertrees methods (other than MRP) to generate starting trees might result in a better global strategy to compute RF supertrees and this should be investigated further. We note that the ideas presented in [51] can be directly used to perform efficient NNI-based local searches for the RF supertree problem. In particular, we can show that heuristic searches for the RF supertree problem, which perform a total of *p *local search steps based on 1, 2, or 3-NNI neighborhoods (see [51]), can all be executed in *O*(*kn*(*n *+ *p*)) time; yielding speed-ups of Θ(min{*n, p*}), Θ(*n*·min{*n, p*}) and Θ(*n*^2^·min{*n, p*}) for heuristic searches that are based on naïve algorithms for 1, 2 and 3-NNI local searches respectively. It would also be interesting to see if heuristics based on TBR perform significantly better than those based on SPR in inferring RF supertrees.

In some cases it might be desirable to remove the restriction that the supertree be binary. In the consensus setting, such a median tree can be obtained within polynomial time [27]; however, finding a median RF tree in the supertree setting is NP-hard [52]. One simple way to estimate a non-binary median tree could be to first compute an RF supertree and then to iteratively (and perhaps greedily) contract those edges in the supertree that result in a reduction in the total RF distance. Thus, our algorithms may even be useful for roughly estimating majority-rule(-) supertrees [28], which are essentially the strict consensus of all optimal, not necessarily binary, median RF trees, and have several desirable properties [29]. These majority-rule(-) supertrees are also the strict consensus of all maximum-likelihood supertrees [53]. Also, there are several alternate forms of the RF distance metric that could be incorporated into our local search algorithms. For example, in order to account for biases associated with the different sizes of input trees, we could normalize the RF distance for each input tree, dividing the observed RF distance by the maximum possible RF distance based on the tree size. Similarly, we could incorporate either branch length data or phylogenetic support scores (bootstrap values or posterior probabilities) from the input trees into the RF distance in order to give more weight to partitions that are strongly supported or separated by long branches (e.g., [25,54]). Our current implementation essentially treats all branch lengths as one and all partitions as equal. The addition of branch length or support data may further improve the accuracy of the RF supertree method.

## Competing interests

The authors declare that they have no competing interests.

## Authors' contributions

MSB was responsible for algorithm design and program implementation, contributed to the experimental evaluation, and wrote major parts of the manuscript. JGB performed the experimental evaluation and the analysis of the results, and contributed to the writing of the manuscript. OE and DFB supervised the project and contributed to the writing of the manuscript. All authors read and approved the final manuscript.
